# The complete mitochondrial genome of the zebra seabream *Diplodus cervinus* (Perciformes, Sparidae) from the Mediterranean Sea

**DOI:** 10.1080/23802359.2022.2145174

**Published:** 2022-11-23

**Authors:** David Osca, Luigi Caputi, Valentina Tanduo, Rosa Maria Sepe, Assunta Liberti, Francesco Tiralongo, Iolanda Venuti, Marina Ceruso, Fabio Crocetta, Paolo Sordino, Tiziana Pepe

**Affiliations:** a Department of Integrative Marine Ecology, Stazione Zoologica Anton Dohrn, Naples, Italy; b Department of Biology and Evolution of Marine Organisms, Stazione Zoologica Anton Dohrn, Naples, Italy; c Department of Biological, Geological and Environmental Sciences, University of Catania, Italy; d Scientific Organization for Research and Conservation of Marine Biodiversity, Ente Fauna Marina Mediterranea, Avola, Italy; e Department of Veterinary Medicine and Animal Production, University of Naples "Federico II", Naples, Italy; f Department of Biology and Evolution of Marine Organisms, Stazione Zoologica Anton Dohrn, Sicily Marine Centre, Messina, Italy

**Keywords:** Mitogenome, gene order, base composition, demersal fishes, phylogeny

## Abstract

The complete nucleotide sequence of the mitochondrial (mt) genome of the demersal zebra seabream *Diplodus cervinus* (Lowe, 1838) was determined for the first time. The double stranded circular molecule is 16,559 base pairs (bp) in length and encodes for the typical 37 metazoan mitochondrial genes, and 2 non-coding regions (D-loop and L-origin). The gene arrangement of the *D. cervinus* mt genome follows the usual one for fishes. The nucleotide sequences of the mt protein coding and ribosomal genes of *D. cervinus* mt genome were aligned with orthologous sequences from representatives of the Sparidae family and phylogenetic relationships were inferred. Maximum likelihood analyses placed *D. cervinus* as a sister species of *Diplodus sargus* (Linnaeus, 1758).

The zebra seabream *Diplodus cervinus* (Lowe, 1838) is a gregarious demersal marine fish of the family Sparidae Rafinesque, 1818, usually living in groups of 4–5 individuals over rocky bottoms up to 80 m depth, although it can be also found on muddy bottoms up to 300 m (Bauchot and Hureau 1986; Pajuelo, Lorenzo, and Domínguez-Seoane 2003). This thermophilic species is distributed in the eastern Atlantic Ocean from the Bay of Biscay to Cape Verde Islands, from Angola to South Africa, and around Madeira and Canary Islands, but it also occurs in the Mediterranean Sea, where it is recently widening its distribution (Bauchot and Hureau 1986; Pajuelo, Lorenzo, and Domínguez-Seoane 2003; Tiralongo et al. 2020). It reaches ∼55 cm in length and 2.7 kg in weight, and it is a species of interest in small scale fisheries throughout its range of distribution, with scattered attempts to rear it using aquaculture techniques (Bauchot and Hureau 1986; IGFA 2001). This species, together with other ones of the genus *Diplodus*, represents a candidate with great potential for aquaculture, due to its market price and good adaptability to farming environment (Coutinho et al. 2016). In some coastal areas, like those of Canary Islands, this demersal species covers a relevant ecological role (Pajuelo, Lorenzo, Domínguez, et al. 2003).

The *D. cervinus* specimen analyzed in this study was meant for sale as seafood to the consumers. The specimen was caught by local fisherman and it was collected for research as a dead specimen from the fisherman’s market (36.7406 N, 15.1193E), where it was supplied directly from local fishermen. Thus, it did not undergo any manipulation or experimentation in the laboratory. Its usage for scientific purposes is not included in the Article 2 of the Italian Legislative Decree n. 26 of 4 March 2014, national transposition of the European Directive 2010/63/UE. Complete mt genome sequence of *D. cervinus* and its annotation is presented here for the first time. The specimen was caught with trammel nets (1.5 mt depth) on a rocky bottom off Marzamemi (Syracuse, Sicily, Italy; Ionian Sea, Mediterranean Sea, ∼36.7480 N, 15.1129E) on 18 April 2020. It was morphologically identified based on species-specific diagnostic characters and subsequently deposited in the Darwin Dohrn Museum of the Stazione Zoologica Anton Dohrn of Naples (http://193.205.231.138/ZooColl/HTML/index.php, curator Andrea Travaglini, andrea.travaglini@szn.it) with the code number SZN-OST-0003. Total DNA was extracted from 25 mg of dorsal muscle tissue following Mascolo, Ceruso, Sordino, et al. (2019) methodology. The assembled and annotated mitogenome was obtained by high-throughput sequencing of enriched mitochondrial DNA with Illumina NextSeq 550 System (Illumina, San Diego, CA, USA). The obtained sequences were assembled using MegaHit (Li et al. 2015) through the Galaxy server at https://usegalaxy.eu/ (Afgan et al. 2018). The final contig obtained was annotated by the MitoFish server (Iwasaki et al. 2013) and subsequently checked manually.

The *D. cervinus* mitogenome was 16,559 bp long. The overall nucleotide composition was: 27.58% A, 29.30% C, 26.56% T, and 16.56% G, being similar to other Sparidae mitogenome data (Ceruso et al. 2018a, 2018b, 2020; Mascolo et al. 2018a, 2018b; Mascolo, Ceruso, Chirollo, et al. 2019; Caputi et al. 2021). As is the case for most metazoans (Boore 1999), the mtDNA encoded for 13 protein-coding genes, 22 tRNAs, and 2 rRNAs. Also, two non-coding regions (D-loop and L-origin) were present, in agreement with fish mitochondrial genomes (Satoh et al. 2016). The heavy strand of the mt genome encoded for 12 protein-coding genes, the majority of the tRNA genes, and the 2 ribosomal genes (*12S* and *16S*). The *NADH dehydrogenase subunit 6* (*nad6*) gene and the *trnA*, *trnN*, *trnC*, *trnY*, *trnE*, and *trnC* were encoded by the light strand. The mt genome organization followed those previously described (see Ceruso et al. 2020; Fietz et al. 2020; Caputi et al. 2021).

All protein-coding genes started with the codon ATG except for the subunit 1 of the cytochrome oxidase (*cox1*) that started with GTG. Some genes had complete stop codons (TAA in *nad4L* and *nad5*; TAG in subunit 8 of the ATP synthase (*atp8*), *nad1* and *nad6*; AGG in *cox1*), while other genes ended with a single T (*cox2*, *cob*, *nad3*, and *nad4*) or TA (*atp6*, *cox3*, and *nad2*), which presumably becomes functional by subsequent polyadenylation of the transcribed messenger RNA (Ojala et al. 1981).

A maximum likelihood (ML) analysis was implemented to elucidate the phylogenetic position of *D. cervinus.* It was performed in RAxML-NG (Kozlov et al. 2019). The resultant phylogeny (Figure 1) placed *D. cervinus* as sister species of *D. sargus* (Linnaeus, 1758) with maximum support, and both species as sister group of *Diplodus puntazzo* (Walbaum, 1792), again with maximum support. So, all three latter species formed a monophyletic group that corresponded to a clade including the species of the genus *Diplodus* Rafinesque, 1810. This clade was placed in the topology as a sister group of a clade including the species of the genus *Acanthopagrus* Peters, 1855, with a ML support of 94. This last clade was recovered as sister group of *Pagellus acarne* (Risso, 1827) and *Pagellus bogaraveo* (Brünnich, 1768), similar to previous studies (Ceruso et al. 2020; Caputi et al. 2021).

**Figure 1.**
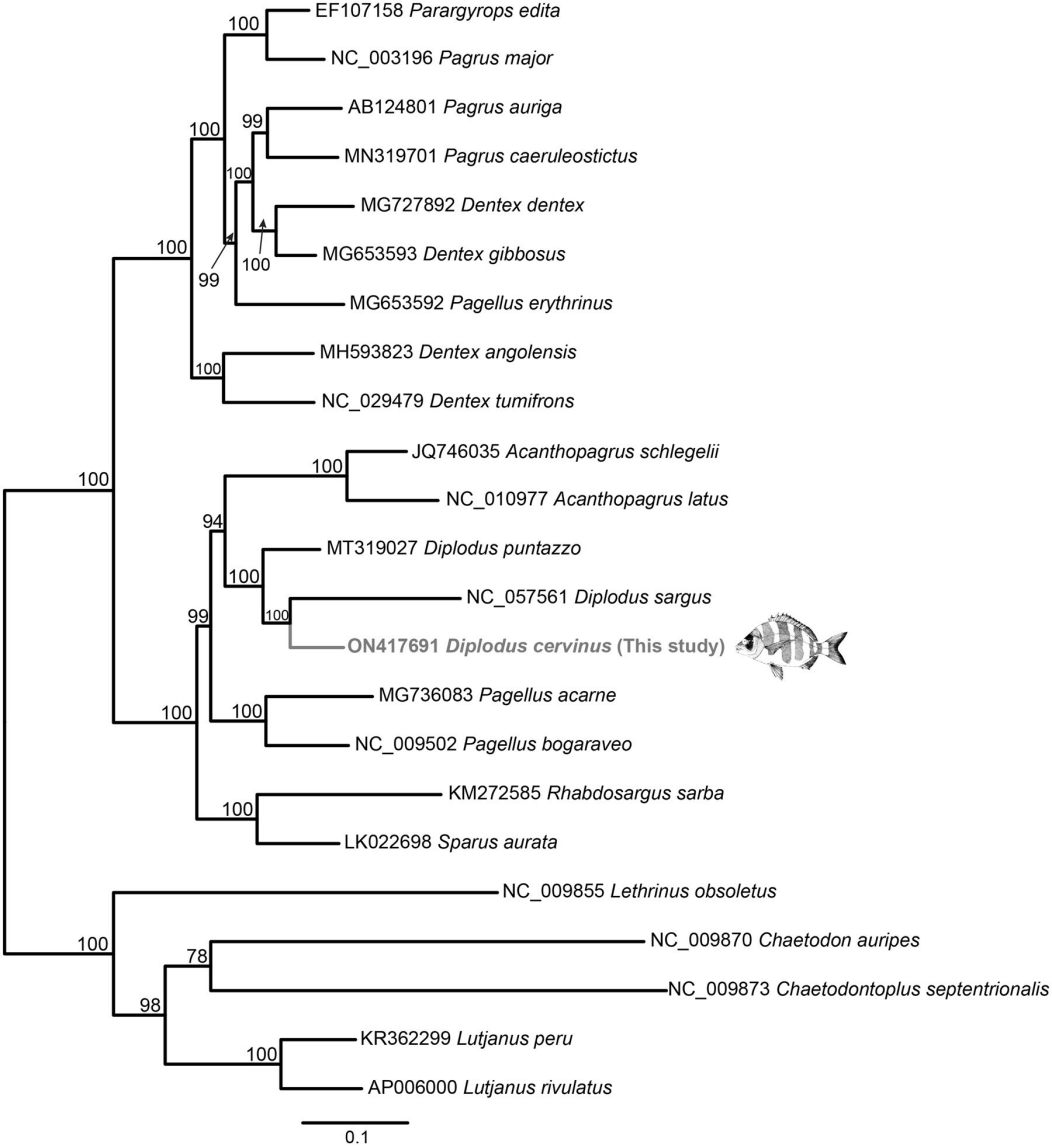
Phylogenetic relationships in the family Sparidae based on the complete mt genome sequences available in GenBank and that of *Diplodus cervinus* reported here (*Acanthopagrus latus* NC_010977; *Acanthopagrus schlegelii* JQ746035; *Dentex angolensis* MH593823; *Dentex* MG727892; *Dentex gibbosus* MG653593; *Dentex tumifrons* NC_029479; *Diplodus puntazzo* MT319027; *Diplodus sargus* NC_057561; *Pagellus acarne* MG736083; *Pagellus bogaraveo* NC_009502; *Pagellus erythrinus* MG653592; *Pagrus auriga* AB124801; *Pagrus caeruleostictus* MN319701; *Pagrus major* NC_003196; *Parargyrops edita* EF107158; *Rhabdosargus sarba* KM272585; *Sparus aurata* LK022698). Five outgroup species (*Lutjanus peru* KR362299, *Lutjanus rivulatus* AP006000, *Lethrinus obsoletus* NC_009855, *Chaetodontoplus septentrionalis* NC009873, and *Chaetodon auripes* NC_009870) were selected. Maximum likelihood method was used with an automatic bootstrapping cutoff of 0.01.

## Data Availability

The data that support the findings of this study are openly available in GenBank of NCBI at [https://www.ncbi.nlm.nih.gov] under the accession no. ON417691. The associated BioProject, SRA, and Bio-Sample numbers are PRJNA836136, SRR19136359, and SAMN28132046, respectively.
